# Increasing Radiation Dose to the Thoracic Marrow Is Associated With Acute Hematologic Toxicities in Patients Receiving Chemoradiation for Esophageal Cancer

**DOI:** 10.3389/fonc.2019.00147

**Published:** 2019-03-15

**Authors:** Denise Fabian, Ahmet Ayan, Dominic DiCostanzo, Christian L. Barney, Jihad Aljabban, Dayssy A. Diaz, Eric D. Miller, Evan Wuthrick, Terence M. Williams, Jose G. Bazan

**Affiliations:** ^1^Department of Radiation Oncology, The Ohio State University Comprehensive Cancer Center, Arthur G. James Cancer Hospital and Richard J. Solove Research Institute, Columbus, OH, United States; ^2^Methodist East Brook Cancer Center, Omaha, NE, United States; ^3^Department of Radiation Oncology, Moffitt Cancer Center, Tampa Bay, FL, United States

**Keywords:** hematologic toxicity, radiation dose, bone marrow, esophageal cancer, IMRT, 3DCRT, NTCP

## Abstract

**Purpose:** To test the hypothesis that increasing radiation dose to the thoracic marrow (TM) contributes to the development of hematologic toxicities (HT) in esophageal cancer (EC) patients receiving chemoradiation therapy (CRT).

**Methods:** We identified EC cases treated with curative intent CRT at our institution from 2007 to 2016. The TM was contoured as the union of the vertebral bodies (VB) from T1-L1, the ribs from T1-L1, and the sternum. The TM-mean dose and the TM volume receiving at least 5–50 Gy (V5-V50) were collected. Grade ≥ 3 HT (HT3+) was the primary endpoint. Normal tissue complication probability (NTCP) was evaluated using the Lyman-Kutcher-Burman (LKB) model. Logistic regression was used to test associations between HT3+ and dosimetric parameters. Odds ratios (OR) and 95% confidence intervals (CI) are reported with *p* < 0.05 considered significant. Receiver operating characteristics analysis was used to determine optimal cut points.

**Results:** We identified 137 EC cases, and most received concurrent carboplatin/paclitaxel (*N* = 83). Median radiation dose was 50.4 Gy (IQR = 50.4–50.4 Gy). The rate of HT3+ was 39.4%. Optimization of the LKB model yielded the results *n* = 0.70, *m* = 0.67, and TD_50_ = 20.1 Gy. The TM-V30 was most strongly associated with HT3+ and on multivariate analysis, patients with TM-V30 ≥ 14% had a 5.7-fold (95% CI 2.42–14.54, *p* < 0.001) increased odds of HT3+ in the entire cohort and a 4-fold (95% CI 1.54–11.11, *p* = 0.006) increased odds of HT3+ in the carboplatin/paclitaxel cohort compared to patients with TM-V30 < 14%. Radiation dose to the VB and rib sub-sites of the TM were also associated with HT3+, particularly VB-V40.

**Conclusion:** We found that increasing TM radiation dose was associated with HT3+ in EC patients treated with CRT. Radiation dose to the VB and rib sub-sites were also associated with HT3+. These findings suggest that limiting radiation dose to the TM (or its sub-sites) may be sufficient to decrease HT3+, but further prospective evaluation of these results is needed.

## Introduction

Esophageal cancer is a common malignancy and leads to 16,000 deaths in the United States each year (1). Most patients with locally-advanced esophageal cancer are treated with concurrent chemoradiation (CRT) in the neoadjuvant or definitive setting (2, 3). CRT is often associated with severe acute hematologic toxicities (HT) (4, 5).

Myelosuppression is a negative prognostic factor in patients undergoing CRT for esophageal cancer (4, 6). Despite this, little is understood about the additional myelosuppressive impact of radiation to the bony structures in the thorax, such as the thoracic vertebral bodies (VB), when given with chemotherapy. Previous reports have suggested that greater radiation (RT) doses to the thoracic VB during CRT for lung cancer are associated with greater rates of HT (7, 8). In 2016, a report of 46 esophageal cancer patients receiving CRT with cisplatin and 5-fluorouracil found greater thoracic VB and rib irradiation to be associated with grade 3 leukopenia (9). Therefore, efforts to reduce radiation dose to the thoracic marrow (TM) may lead to reductions in HT. The potential benefits of reducing HT include but are not limited to: less frequent therapy interruptions or delays in care, decreased hospitalizations, decreased need for transfusions, decreased risk of infection and decreased use of antibiotics, improved quality of life, reduced costs of care, and importantly, improving cancer control/disease outcome. In the era of intensity modulated radiation therapy (IMRT), reducing the dose to the TM is potentially achievable.

In this study we aim to determine the impact of radiation dose to the TM on the development of HT. We hypothesize that increasing radiation dose to the TM contributes to the development of grade ≥ 3HT (HT3+) in esophageal cancer patients receiving CRT.

## Materials and Methods

### Patient Selection

We identified patients with histologically confirmed esophageal adenocarcinoma and squamous cell carcinoma treated with curative intent either neoadjuvant or definitive CRT at our institution between 2007 and 2016 in this institutional review board-approved, retrospective study. Patients were required to have complete blood count (CBC) data, which included a baseline CBC (at least 1 week prior to start of CRT) and at least 3 values during treatment. Patients who received induction chemotherapy (IC) were included in this study, provided blood counts recovered before CRT initiation. Initially, 162 patients were identified who completed radiation therapy at our institution. There were 21 patients with incomplete CBC data and 3 additional patients who did not recover blood counts after IC that were excluded from the study. One patient did not have a planning CT scan that included all the vertebral bodies contoured in our study and was thus excluded. The remaining 137 patients formed the cohort for this study.

### Radiation Therapy

A free-breathing CT scan was used for patient simulation. Patients were simulated in the supine position with arms over head. A vacuum cushion bag was utilized for immobilization. The gross tumor volume (GTV) was contoured on the CT scan, encompassing the primary tumor. The gross nodal volume (GTVn) was contoured to include all identifiable nodal disease. When available, PET fusion was used to help identify areas of active disease. Expansion of the GTV and GTVn was performed to create the clinical target volume (CTV) in order to include microscopic disease, and the CTV further expanded to a planning target volume (PTV), to account for clinical set-up error. Radiation was delivered at doses of 1.8–2.0 Gy per fraction to the PTV to a dose of 50.4–59.4 Gy utilizing 3-D conformal (3DRT) or IMRT techniques.

### Chemotherapy

Chemotherapy regimens included carboplatin/paclitaxel (typically carboplatin AUC 2 mg/m^2^/min and paclitaxel 50 mg/m^2^), cisplatin/5-fluorouracil (FU) (typically cisplatin 75 mg/m^2^ and 5-FU 1,000mg/m^2^), cisplatin/irinotecan (typically cisplatin 30 mg/m^2^ and Irinotecan 65 mg/m^2^), or 5FU alone (typically 5FU 300 mg/m^2^).

### Data Collection

**Figure 1:**
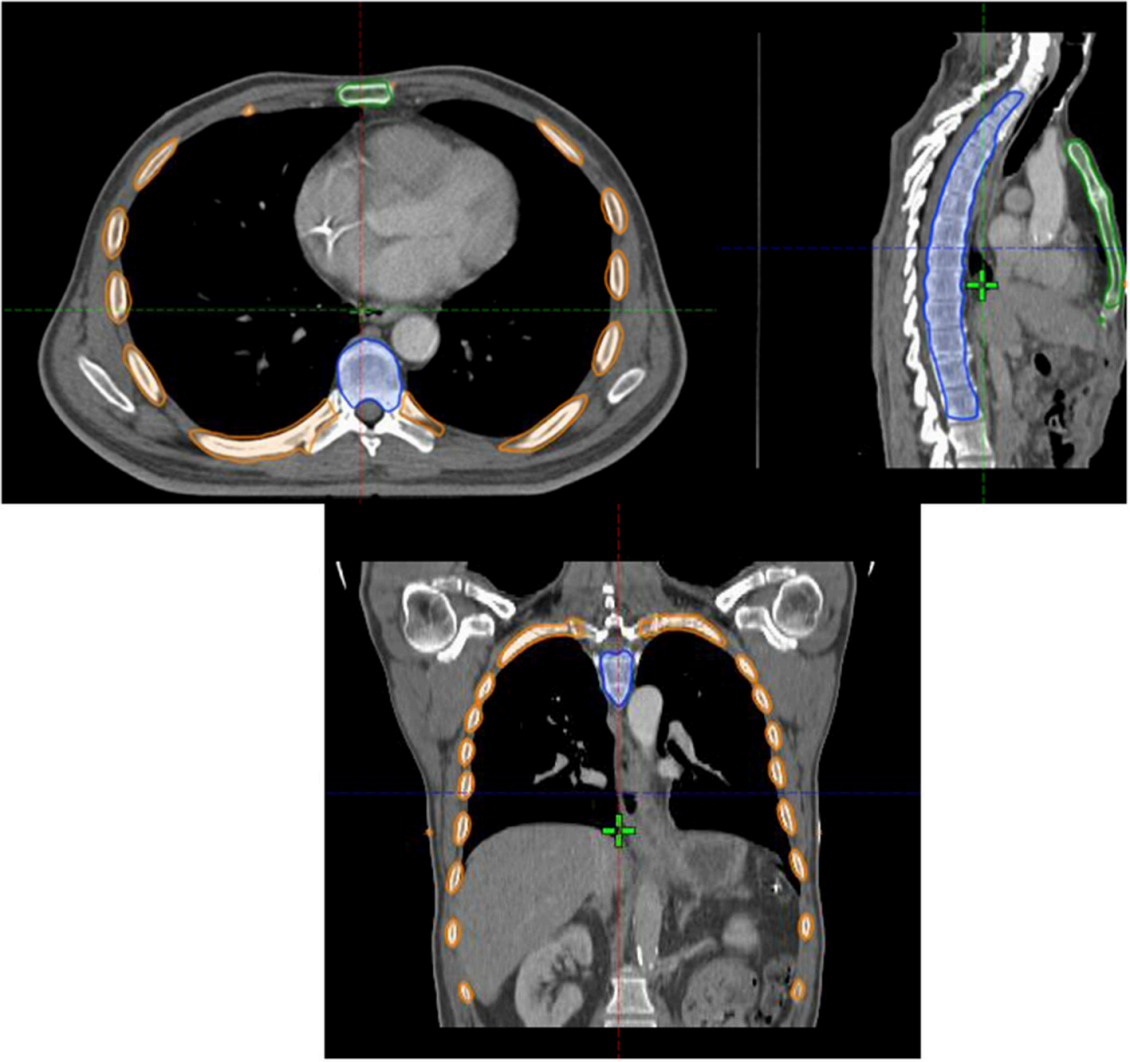
Axial **(top left)**, sagittal **(top right)** and coronal **(bottom)** images of the thoracic marrow structure comprised of the sternum (green contour), ribs (orange contour), and vertebral bodies from T1-L1 (blue contour).

We retrospectively contoured the sternum, the VBs from T1-L1, and the ribs from T1-L1 for all patients (Figure 1). We defined the TM as the union of the sternum, VBs, and ribs. The TM volumes receiving 5–50 Gy (V5-V50) along with the mean TM doses were calculated from the dose volume histogram (DVH). This was done for each of the three TM sub-sites as well. CBC data was obtained from the electronic medical record. The nadirs for absolute white blood count (WBC), neutrophil count (ANC), and absolute platelet count were recorded. Hemoglobin was not included given that EC patients may present with anemia. The Common Terminology Criteria for Adverse Events version 5 was used to grade the hematologic nadir. The primary endpoint analyzed was HT3+ for WBC, ANC, and/or absolute platelet count.

### Statistics

Normal tissue complication probability (NTCP) was evaluated with a simplified Lyman-Kutcher-Burman (LKB) model for HT3+ using the TM as the organ-at-risk (OAR), as previously described (7, 10, 11). Maximum likelihood estimations (MLE) were used to determine optimal values for the three parameters defined by the LKB model; specifically, *n*, the volume parameter; *m*, the slope parameter; and *TD*_50_, the uniform OAR dose which results in a 50% complication risk.

Differences in baseline dosimetric parameters between the IMRT and 3DRT groups were evaluated by the Wilcoxon test. Univariate and multivariate analyses were performed using logistic regression to determine the correlation of TM V_5_-V_50_ and TM mean dose with HT3+. This was done for each of the 3 sub-sites as well. All clinical and dosimetric parameters with *p* ≤ 0.10 on univariate analysis were included in the multivariate model, with each dosimetric parameter being evaluated independently. Receiver operating characteristic (ROC) analysis was used to determine the optimal cut-point for dosimetric parameters. MATLAB version 9.0 (The MathWorks, Natick MA) and R version 3.3.1 (R Foundation for Statistical Computing, Vienna) optimization routines were used to maximize likelihood NTCP parameters. All other statistical analyses were performed using SAS version 9.4 (SAS Institute, Cary, NC).

## Results

### Summary of Patient Characteristics and TM Dosimetric Parameters

Patient, tumor, and radiation treatment characteristics are described in Table 1. The cohort included 137 patients that were mostly male (84%), median age 61 years (Interquartile range [IQR], 55–67), and predominantly with clinical stage II (34%) or stage III (54%) disease. Over 80% of tumors were located in the lower thoracic (*N* = 66) or gastroesophageal junction (*N* = 49). Most patients received concurrent carboplatin/paclitaxel (*N* = 83) while the rest received platinum/5FU (*N* = 28), irinotecan/platinum (*N* = 22), or 5FU alone (*N* = 4). A total of 43 (32%) patients received induction chemotherapy. Median RT dose was 50.4 Gy (IQR = 50.4–50.4 Gy), and the majority of patients were treated with 3DCRT (63.5%). Most patients underwent surgery after neoadjuvant CRT (60.6%).

**Table 1:** Baseline patient characteristics (*N* = 137).

Age, mean (*SD*)	61.0 years (*SD* = 11.3)
Gender, *n* (%)	Male: 115 (84%) Female: 22 (16%)
BMI, mean (*SD*)	27.5 kg/m^2^ (7.2)
**TUMOR LOCATION, *n* (%)**	
Upper/Middle Thoracic	14 (10.2%)
Lower Thoracic	66 (48.2%)
GE Junction	49 (35.8%)
Not specified	8 (5.8%)
**HAD SURGERY**	
Yes	83 (60.6%)
No	54 (39.4%)
**T STAGE, *n* (%)**	
T1 or T2	28 (20.4%)
T3 or T4	102 (74.5%)
TX	7 (5.1%)
**N STAGE, *n* (%)**	
N0	36 (26.3%)
N+	95 (69.3%)
Nx	6 (4.4%)
**AJCC STAGE, *n* (%)**	
Stage I	5 (3.6%)
Stage II	46 (33.7%)
Stage III	74 (54.0%)
Stage IV	7 (5.1%)
Unknown Stage	5 (3.6%)
**CHEMOTHERAPY REGIMEN**	
Carboplatin/paclitaxel	83 (60.6%)
Platinum/5FU	28 (20.4%)
Platinum/Irinotecan	22 (16.1%)
5FU alone	4 (2.9%)
RT Dose, median (IQR)	50.4 Gy (50.4–50.4 Gy)
RT Fractions, median (IQR)	28 (28–28)
**RT TECHNIQUE, *n* (%)**	
3DRT	87 (63.5%)
IMRT	50 (36.5%)

*SD, standard deviation; BMI, Body Mass Index; IQR, interquartile range; AJCC, American Joint Committee on Cancer; 5FU, 5-fluorouracil; RT, Radiation Therapy; 3DRT, 3D conformal radiation therapy; IMRT, intensity-modulated radiation therapy*.

Summary statistics for the TM mean dose and TM V5-V50 are summarized in Table 2. The TM mean dose had a median value of 13.0 Gy (IQR, 11.1 Gy−15.3 Gy). Patients treated with IMRT (*N* = 50) had significantly higher TM V5 (60.5% vs. 52.4%, *p* = 0.001) and TM V10 (46.0% vs. 39.7%, *p* = 0.001) compared to patients treated with 3DRT. Based on doses to the sub-sites, these differences in V5 and V10 between IMRT and 3DRT patients seem to be driven by dose to the ribs, as shown in Table 2. The TM V30 had a trend toward lower values in the IMRT vs. 3DRT patients (13.4% vs. 17.8%, *p* = 0.07). There were no other significant differences in TM dosimetric parameters between IMRT and 3DRT patients.

**Table 2:** Summary statistics of dosimetric parameters.

**Parameter**	**All patients**	**3DRT**	**IMRT**	***p*-value**
TM-Mean, median (IQR)	13.0 (11.1–15.3) Gy	12.7 (10.8–14.9) Gy	13.4 (11.2–16.0) Gy	0.18
VB	27.9 (22.9–31.7) Gy	27.7 (23.0–32.4) Gy	27.6 Gy (22.0–31.1) Gy	0.43
Ribs	8.6 (7.8–10.3) Gy	8.4 (7.6–9.7) Gy	9.9 (8.1–12.1) Gy	0.002
Sternum	13.5 (6.6–19.6) Gy	13.4 (5.7–20.8) Gy	13.6 (7.6–18.5) Gy	0.973
TM-V5, median (IQR)	55.0% (44.4–66.4%)	52.4% (43.2–60.5%)	60.5% (54.2–76.0%)	0.001
VB	76.6% (67.1–83.1%)	75.0% (55.6–81.5%)	78.5% (66.5–84.0%)	0.30
Ribs	51.0% (39.5–62.1%)	47.1% (37.2–55.7%)	54.8% (48.7–73.7%)	<0.001
Sternum	52.5% (36.7–80.1%)	50.3% (35.3–70.1%)	60.3% (40.2–97.8%)	0.044
TM-V10, median (IQR)	42.0% (35.0–49.2%)	39.7% (33.0–44.8%)	46.0% (38.4–59.3%)	0.001
VB	73.7% (63.9–80.5%)	71.6% (65.1–79.5%)	75.2% (63.6–82.2%)	0.32
Ribs	31.6% (25.4–41.3%)	29.3% (23.5–37.8%)	38.8% (28.8–53.5%)	<0.001
Sternum	47.0% (25.0–73.4%)	46.7% (21.0–63.2%)	50.6% (28.8–81.7%)	0.13
TM-V20, median (IQR)	26.9% (21.4–30.8%)	26.9% (21.6–30.1%)	26.5% (20.9–35.9%)	0.62
VB	69.7% (56.6–77.9%)	68.8% (58.2–77.3%)	71.0% (53.8–78.9%)	0.68
Ribs	14.6% (10.0–18.8%)	14.4% (10.3–17.7%)	15.1% (9.2–20.5%)	0.41
Sternum	30.6% (3.6–50.2%)	34.2% (2.9–52.3%)	25.0% (5.0–48.9%)	0.71
TM-V30, median (IQR)	15.3% (11.0–22.1%)	17.8 (11.1–22.7%)	13.4% (10.9–17.5%)	0.07
VB	56.0% (42.2–68.6%)	58.7% (42.9–72.0%)	52.1% (35.0–64.1%)	0.07
Ribs	4.0% (0.9–8.4%)	6.1% (1.1–8.4%)	1.9% (0.6–8.5%)	0.12
Sternum	3.2% (0.0–31.0%)	11.1% (0.0–44.8%)	0.9% (0.0–17.2%)	0.02
TM-V40, median (IQR)	6.8% (4.7–11.2%)	6.9% (4.7–11.3%)	6.0% (4.3–9.1%)	0.37
VB	29.7% (19.3–46.6%)	31.5% (20.4–49.4%)	27.1% (16.7–36.6%)	0.06
Ribs	0.1% (0.0–1.5%)	0.2% (0.0–0.5%)	0.0% (0.0–2.0%)	0.69
Sternum	0.0% (0.0–0.0%)	0.0% (0.0–0.6%)	0.0% (0.0–0.0%)	0.69
TM-V50, median (IQR)	1.6% (0.6–2.7%)	1.7% (0.7–2.6%)	1.1% (0.4–3.0%)	0.50
VB	6.7% (2.9–12.7%)	7.2% (3.4–13.0%)	6.2% (1.8–10.3%)	0.30
Ribs	0.0% (0.0–0.0%)	0.0% (0.0–0.0%)	0.0% (0.0–0.0%)	0.69
Sternum	0.0% (0.0–0.0%)	0.0% (0.0–0.0%)	0.0% (0.0–0.0%)	0.53

*3DRT, 3D conformal radiation therapy; IMRT, intensity-modulated radiation therapy; TM, thoracic marrow; IQR, interquartile range; VB, vertebral body dose*.

### Clinical Hematologic Toxicities

Overall grade 3+ hematologic toxicity was 39.4% and overall grade 2+ toxicity was 73.0% (Table 3). The median time to grade 3+ toxicity was 28 days (IQR = 21–32 days). Incidence of Grade 3+ toxicity varied by systemic therapy regimen and was 48.2% (40 of 83 patients), 40.9% (9 of 22 patients), and 17.9% (5 of 28 patients) for those who were treated with carboplatin/paclitaxel, irinotecan-based chemotherapy, and platinum/5FU-based chemotherapy, respectively.

**Table 3:** Acute hematologic toxicities observed in cohort.

**Toxicity grade**	**WBC**	**ANC**	**Plt**
Grade 1	38 (27.7%)	84 (61.3%)	114 (83.2%)
Grade 2	48 (35.0%)	25 (18.3%)	13 (9.5%)
Grade 3	44 (32.1%)	25 (18.3%)	10 (7.3%)
Grade 4	7 (5.1%)	3 (2.1%)	0 (0%)
Overall Grade 2+ Toxicity: 100 (73.0%)			
Overall Grade 3+ Toxicity: 54 (39.4%)			

*WBC, white blood cell count; ANC, absolute neutrophil count; PLT, platelet count*.

In total, 10 patients had chemotherapy dose reduction [1 for neutropenic fever, 1 for nausea requiring percutaneous endoscopic gastrostomy tube (PEG) placement, 1 for low ANC, and 1 for thrush, mucositis, nausea/vomiting, and weight loss, 1 for nausea, 1 for mucositis, 1 for poor performance status at start of treatment, 2 for age, 1 for diarrhea]. Also, 20 patients were hospitalized during treatment for various reasons including: dehydration, dysphagia, failure to thrive, nausea, vomiting, pneumonia, fever, and neutropenia. Chemotherapy interruption due to hematologic toxicity was seen in 17 patients whereas chemotherapy discontinuation due to hematologic toxicity was required in 4 patients.

### LKB NTCP Modeling

**Figure 2:**
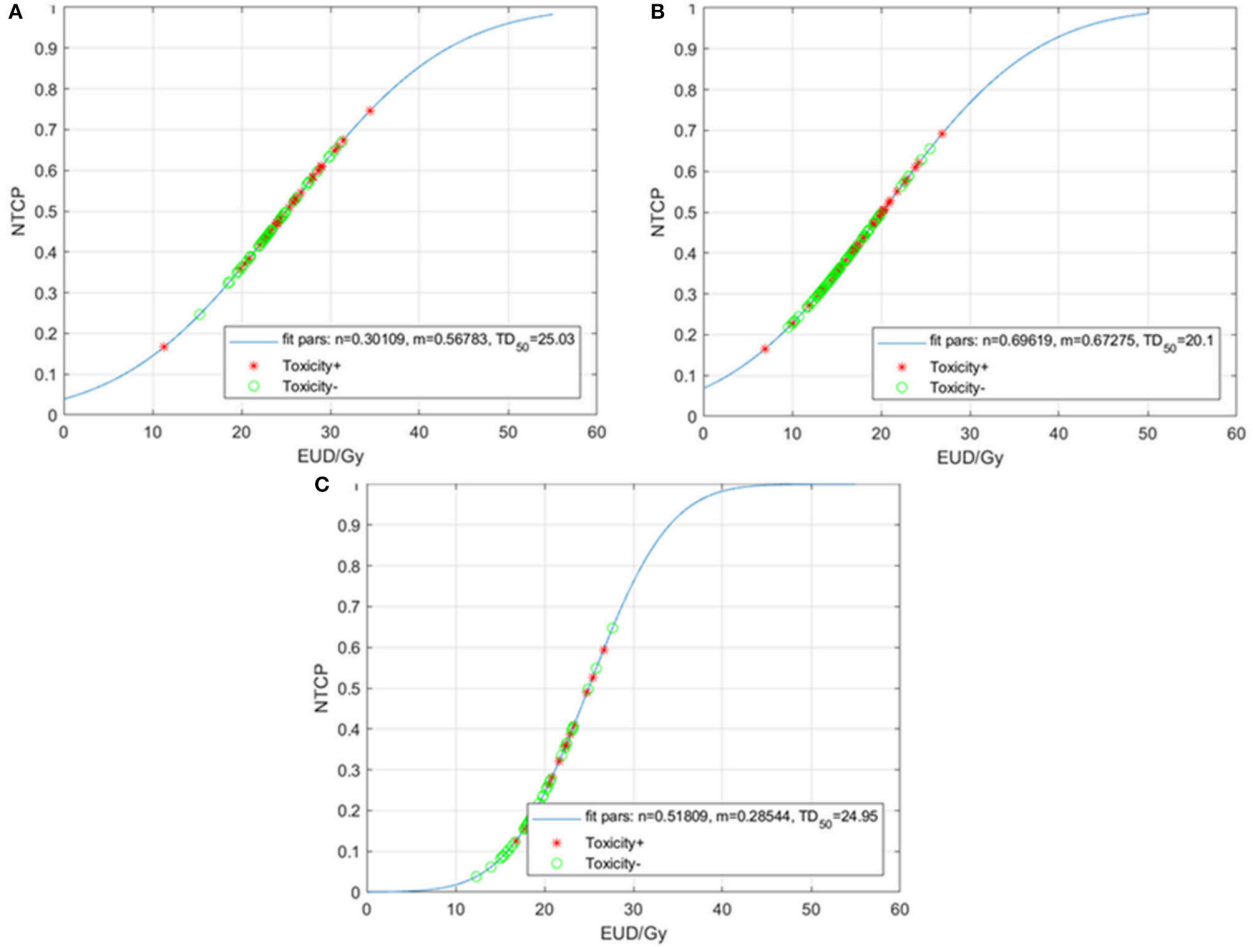
Normal tissue complication probability (NTCP) for acute grade ≥3 hematologic toxicities (HT3+) per the Lyman-Kutcher Burman model for all patients **(A)**, patients treated with carboplatin/paclitaxel **(B)**, and those treated with other systemic therapy regimens **(C)**. Patients that experienced HT3+ during chemoradiation are represented by red asterisks; patients without HT3+ are represented by green circles. EUD, equivalent uniform dose.

Constrained optimization (0 < *n* ≤ 1, with *m* and TD_50_ unrestricted) of the LKB model for HT3+ for the entire cohort resulted in MLE values of *n* = 0.70, *m* = 0.67, and TD_50_ = 20.1 Gy (Figure 2A). In terms of the sub-sites, the resulting values of *n, m*, and TD_50_ were 0.09, 0.24, and 46.3 Gy for the VB; 0.60, 0.56, and 14.4 Gy for the ribs; and 2, 1.81, and 24.5 Gy for the sternum.

Given the heterogeneity of the chemotherapy regimens, we performed separate analyses for the patients treated with carboplatin/paclitaxel (*N* = 83) and for those treated with other regimens (*N* = 54). The LKB model for HT3+ in the carboplatin/paclitaxel group generated MLE values of *n* = 0.30, *m* = 0.57, and TD_50_ = 25.0 Gy (Figure 2B) compared to *n* = 0.51, *m* = 0.28, and TD_50_ = 25.0 Gy (Figure 2C).

**Figure 3:**
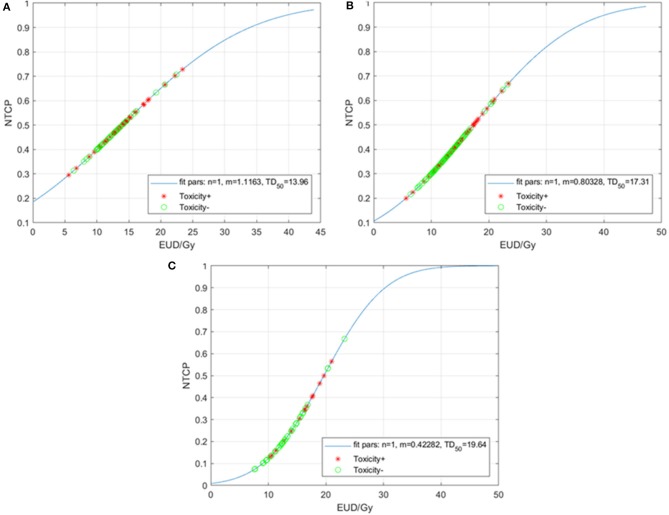
Normal tissue complication probability (NTCP) for acute grade ≥3 hematologic toxicities (HT3+) per the Lyman-Kutcher Burman model with *n* fixed (*n* = 1) for all patients **(A)**, patients treated with carboplatin/paclitaxel **(B)**, and those treated with other systemic therapy regimens **(C)**. Patients that experienced HT3+ during chemoradiation are represented by red asterisks; patients without HT3+ are represented by green circles. EUD, equivalent uniform dose.

Last, we performed a further exploratory analysis where *n* was fixed at the value *n* = 1, as this forces the model to treat the TM as a parallel (rather than serial) OAR. Figure 3 summarizes the results. For the entire cohort, the resulting MLE values are *m* = 0.80, and TD_50_ = 17.3 Gy (Figure 3A). When separated by type of chemotherapy, the *m* and TD_50_ values are 1.12 and 14.0 Gy for the carboplatin/paclitaxel group (Figure 3B) compared to 0.42 and 19.6 Gy for patients treated with other regimens (Figure 3C).

### Predictors of Hematologic Toxicities: Total Marrow

On univariate analysis, patient age (OR = 1.04, *p* = 0.028), male gender (OR = 0.38, *p* = 0.044), and systemic therapy regimen (carboplatin/paclitaxel vs. other, OR = 2.66, *p* = 0.010) were factors significantly associated with the odds of HT3+, while body mass index (BMI), IMRT (vs. 3DRT), and use of induction chemotherapy were not (Table 4). In terms of dosimetric predictors, increasing TM mean dose (per Gy) was associated with significantly higher rates of HT3+ (OR = 1.13, 95% CI 1.02–1.26, *p* = 0.021). In addition, each 5% increase in TM-V30 was associated with higher rates of HT3+ (OR = 1.31, 95% CI 1.05–1.65, *p* = 0.019) with similar results seen for TM-V50 (OR = 3.10, 95% CI 1.13–9.12, *p* = 0.032). The other TM dosimetric parameters (V5, V10, V20, and V40) were borderline (*p* < 0.10) associated with higher rates of HT3+.

**Table 4:** Univariate logistic regression analysis for factors associated with grade ≥ 3 hematologic toxicity in the entire cohort.

**Parameter**	**OR (95% CI), *p*-value**
Age (per year)	1.04 (1.00–1.07), *p* = 0.028
BMI (per kg/m^2^)	1.00 (0.95–1.05), *p* = 0.939
Male vs. Female	0.38 (0.15–0.97), *p* = 0.044
IMRT vs. 3DRT	0.80 (0.39–1.63), *p* = 0.535
Induction chemotherapy vs. No induction	0.86 (0.41–1.81), *p* = 0.686
Carboplatin/paclitaxel vs. Other regimen	2.66 (1.26–5.60), *p =* 0.010
TM-V5	1.10 (0.98–1.24), *p =* 0.099
VB-V5 (per 5% increase)	1.05 (0.97–1.17), *p =* 0.399
Rib-V5	1.09 (0.98–1.22), *p =* 0.117
Sternum-V5	1.07 (1.01–1.15), *p =* 0.026
TM-V10 (per 5% increase)	1.16 (1.00–1.34), *p =* 0.055
VB-V10 (per 5% increase)	1.05 (0.95–1.18), *p =* 0.352
Rib-V10	1.163 (1.01–1.35), *p =* 0.046
Sternum-V10	1.07 (1.01–1.14), *p =* 0.030
TM-V20 (per 5% increase)	1.23 (0.99–1.54), *p =* 0.070
VB-V20 (per 5% increase)	1.07 (0.96–1.19), *p =* 0.225
Rib-V20	1.26 (0.98–1.64), *p =* 0.071
Sternum-V20	1.07 (1.01–1.14), *p =* 0.018
TM-V30 (per 5% increase)	1.31 (1.05–1.65), *p =* 0.019
TM-V30 ≥ 14%	2.95 (1.43–6.11), *p =* 0.003
VB-V30 (per 5% increase)	1.09 (1.00–1.20), *p =* 0.055
Rib-V30	1.59 (1.11–2.32), *p =* 0.013
Sternum-V30	1.06 (1.00–1.13), *p =* 0.056
TM-V40 (per 5% increase)	1.29 (0.97–1.75), *p =* 0.092
TM-V40 ≥ 5.8%	2.46 (1.21–5.12), *p =* 0.014
VB-V40 (per 5% increase)	1.13 (1.03–1.26), *p =* 0.017
Rib-V40	1.19 (0.61–2.34), *p =* 0.599
Sternum-V40	1.00 (0.90–1.11), *p =* 0.935
TM-V50 (per 5% increase)	3.10 (1.13–9.12), *p =* 0.032
TM-V50 ≥ 1.5%	2.41 (1.20–4.95), *p =* 0.015
VB-V50 (per 5% increase)	1.29 (1.02–1.65), *p =* 0.040
Rib-V50	3.22 (0.02–682.20), *p =* 0.45
Sternum-V50	1.06 (0.49–2.16), *p =* 0.875
TM-mean (per 1 Gy increase)	1.13 (1.02–1.26), *p =* 0.021
TM-mean ≥ 13.7 Gy	2.51 (1.24–5.07), *p =* 0.010
VB-mean (per 5% increase)	1.05 (1.00–1.10), *p =* 0.072
Rib-mean	1.20 (1.04–1.40), *p =* 0.013
Sternum-mean	1.04 (1.00–1.08), *p =* 0.03

*OR, odds ratio; CI, confidence interval; BMI, body mass index; IMRT, intensity modulated radiation therapy; 3DRT, 3D conformal radiation therapy; VB, vertebral body; V5-V50, volume of structure receiving ≥5–50 Gy*.

We performed multivariate analyses that included age, gender, and type of systemic therapy regimen along with either TM-mean (model 1), TM-V5 (model 2), TM-V10 (model 3), TM-V20 (model 4), TM-V30 (model 5), TM-V40 (model 6), and TM-V50 (model 7) with results summarized in Table 5. With the exception of TM-V5 and TM-V10, in all models, the adjusted ORs for the dosimetric variable, age, and systemic therapy were significantly associated with HT3+ while the OR for gender was not. For example, in the model that includes TM-V30, the adjusted ORs for TM-V30, systemic therapy regimen (carboplatin/paclitaxel vs. other), age, and gender (male vs. female) were: 1.50 (1.16–1.99, *p* = 0.003), 4.05 (1.76–9.99, *p* = 0.002), 1.04 (1.01–1.08, *p* = 0.028), and 0.49 (0.17–1.37, *p* = 0.175).

**Table 5:** Multivariate logistic regression analysis for factors associated with grade ≥ 3 hematologic toxicity in the entire cohort.

**Model**	**OR (95% CI)**	***p*-value**
**Model 1: TM-mean**		
TM-mean (continuous)	1.15 (1.04–1.29)	0.012
Age	1.04 (1.00–1.08)	0.038
Male vs. Female	0.50 (0.18–1.36)	0.172
C/T vs. Other	3.18 (1.45–7.37)	0.005
TM-mean ≥ 13.7 Gy	2.59 (1.23–5.58)	0.014
Age	1.04 (1.00–1.07)	0.051
Male vs. Female	0.55 (0.20–1.50)	0.240
C/T vs. Other	3.11 (1.42–7.18)	0.006
**Model 2: TM-V5**		
TM-V5	1.10 (0.97–1.25)	0.136
Age	1.04 (1.00–1.08)	0.036
Male vs. Female	0.52 (0.19–1.39)	0.192
C/T vs. Other	2.74 (1.28–6.13)	0.012
**Model 3: TM-V10**		
TM-V10	1.14 (0.98–1.33)	0.104
Age	1.04 (1.00–1.07)	0.048
Male vs. Female	0.51 (0.18–1.37)	0.183
C/T vs. Other	2.73 (1.27–6.11)	0.012
**Model 4: TM-V20**		
TM-V20	1.29 (1.02–1.65)	0.036
Age	1.04 (1.00–1.08)	0.045
Male vs. Female	0.45 (0.16–1.22)	0.117
C/T vs. Other	3.03 (1.40–6.90)	0.006
**Model 5: TM-V30**		
TM-V30	1.50 (1.16–1.99)	0.003
Age	1.04 (1.01–1.08)	0.028
Male vs. Female	0.49 (0.17–1.37)	0.175
C/T vs. Other	1.05 (1.76–9.99)	0.002
TM-V30 ≥ 14%	5.67 (2.42–14.54)	<0.001
Age	1.05 (1.01–1.09)	0.014
Male vs. Female	0.36 (0.12–1.02)	0.058
C/T vs. Other	4.37 (1.88–10.92)	0.001
**Model 6: TM-V40**		
TM-V40	1.52 (1.08–2.20)	0.021
Age	1.04 (1.00–1.08)	0.021
Male vs. Female	0.57 (0.21–1.55)	0.272
C/T vs. Other	3.68 (1.62–8.93)	0.003
TM-V40 ≥ 5.8%	3.50 (1.60–8.11)	0.002
Age	1.04 (1.01–1.08)	0.024
Male vs. Female	0.45 (0.16–1.25)	0.125
C/T vs. Other	3.51 (1.58–8.24)	0.003
**Model 7: TM-V50**		
TM-V50	3.49 (1.19–11.16)	0.027
Age	1.04 (1.00–1.08)	0.039
Male vs. Female	0.56 (0.20–1.53)	0.260
C/T vs. Other	3.15 (1.44–7.27)	0.005
TM-V50 ≥ 1.5%	2.66 (1.26–5.80)	0.012
Age	1.04 (1.00–1.08)	0.039
Male vs. Female	0.50 (0.18–1.36)	0.172
C/T vs. Other	3.04 (1.39–6.96)	0.007

*OR, odds ratio; CI, confidence interval; TM, thoracic marrow; C/T, carboplatin/paclitaxel; Vx, TM volume receiving ≥x Gy*.

Given the heterogeneity in systemic therapy regimens, we performed an analysis in the patients that received carboplatin/paclitaxel, which represents the largest group (*N* = 83). Table 6 shows that the only variables significantly associated with HT3+ on univariate analysis were age (OR = 1.05, 95% CI 1.01–1.10, *p* = 0.014) and TM-V50 (OR = 4.78, 95% CI 1.16–23.50, *p* = 0.039) while TM-V30 was borderline associated with HT3+ (*p* = 0.066). On multivariate analysis, that included age and the dosimetric parameter, Table 7 shows that the only dosimetric variable significantly associated with HT3+ was TM-V50 (OR = 4.78, 95% CI 1.08–25.61, *p* = 0.049).

**Table 6:** Univariate logistic regression analysis for factors associated with grade ≥ 3 hematologic toxicity in patients treated with carboplatin/paclitaxel (*N* = 83).

**Parameter**	**OR (95% CI), *p*-value**
Age (per year)	1.05 (1.01–1.10), *p =* 0.014
BMI (per kg/m^2^)	1.02 (0.96–1.08), *p =* 0.468
Male vs. Female	0.49 (0.15–1.46), *p =* 0.208
IMRT vs. 3DRT	0.92 (0.38–2.23), *p =* 0.849
Induction chemotherapy vs. No induction	0.70 (0.23–2.04), *p =* 0.517
TM-V5 (per 5% increase)	1.06 (0.92–1.23, *p =* 0.449
VB-V5	1.04 (0.90–1.21), *p =* 0.634
Rib-V5	1.04 (0.91–1.20), *p =* 0.539
Sternum-V5	1.06 (0.98–1.15), *p =* 0.175
TM-V10 (per 5% increase)	1.08 (0.90–1.31), *p =* 0.395
VB-V10	1.05 (0.90–1.22), *p =* 0.550
Rib-V10	1.09 (0.91–1.32), *p =* 0.368
Sternum-V10	1.05 (0.98–1.14), *p =* 0.186
TM-V20 (per 5% increase)	1.21 (0.93–1.61), *p =* 0.170
VB-V20	1.07 (0.93–1.24), *p =* 0.363
Rib-V20	1.44 (1.03–2.06), *p =* 0.038
Sternum-V20	1.05 (0.98–1.14), *p =* 0.195
TM-V30 (per 5% increase)	1.32 (0.99–1.82), *p =* 0.066
VB-V30	1.10 (0.98–1.25), *p =* 0.099
Rib-V30	2.03 (1.18–3.74), *p =* 0.015
Sternum-V30	1.04 (0.95–1.14), *p =* 0.453
TM-V40 (per 5% increase)	1.46 (0.96–2.43), *p =* 0.114
VB-V40	1.15 (1.01–1.32), *p =* 0.048
Rib-V40	2.24 (0.67–12.30), *p =* 0.263
Sternum-V40	1.00 (0.84–1.18), *p =* 0.978
TM-V50 (per 5% increase)	4.78 (1.16–23.50), *p =* 0.039
VB-V50	1.34 (0.98–1.89), *p =* 0.081
Rib-V50	5.00 (0.05–999.9), *p =* 0.177
Sternum-V50	5.00 (0.69–999.9), *p =* 0.336
TM mean (per 1 Gy increase)	1.11 (0.98–1.28), *p =* 0.116
VB-mean	1.05 (1.00–1.10), *p =* 0.149
Rib-mean	1.24 (1.01–1.56), *p =* 0.046
Sternum-mean	1.03 (0.98–1.08), *p =* 0.221

*OR, odds ratio; CI, confidence interval; BMI, body mass index; IMRT, intensity modulated radiation therapy; 3DCRT, 3D conformal radiation therapy; VB, vertebral body; V5-V50, VB volume receiving ≥5–50 Gy*.

**Table 7:** Multivariate logistic regression analysis for factors associated with grade ≥ 3 hematologic toxicity in patients treated with carboplatin/paclitaxel (*N* = 83).

**Model**	**OR (95% CI)**	***p*-value**
**Model 1: TM-V30**		
TM-V30 (continuous)	1.34 (1.00–1.89)	0.066
Age	1.06 (1.01–1.11)	0.014
TM-V30 ≥ 14%	3.99 (1.54–11.11)	0.006
Age	1.07 (1.02–1.12)	0.007
**Model 2: TM-V50**		
TM-V50 (continuous)	4.78 (1.08–25.61)	0.049
Age	1.06 (1.01–1.11)	0.017
TM-V50 ≥ 1.5%	2.68 (1.07–6.97)	0.038
Age	1.06 (1.01–1.11)	0.014

*OR, odds ratio; CI, confidence interval; V30 and V50, VB volume receiving ≥30 Gy and ≥50 Gy*.

**Figure 4:**
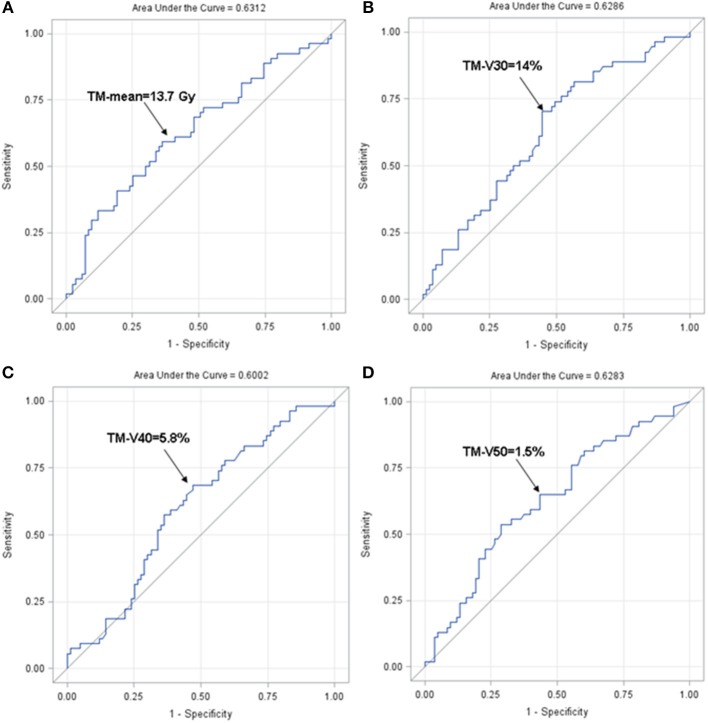
Receiver operating characteristics curve for TM-mean **(A)**, TM-V30 **(B)**, TM-V40 **(C)**, and TM-V50 **(D)**. ROC, receiver operating characteristic.

We further set out to identify the optimal cut-points for the dosimetric variables most strongly associated with HT3+ (TM-mean, TM-V30, TM-V40, and TM-V50) using ROC analysis. Based on the ROC analysis, the optimal cut-points and area under the curve (AUC) for these parameters were 13.7 Gy with AUC = 0.631 (TM-mean), 14% with AUC = 0.629 (TM-V30), 5.8% with AUC = 0.600 (TM-V40), and 1.5% with AUC = 0.628 (TM-V50) (Figure 4). Tables 5, 7 summarize the univariate and multivariate results of these parameters using the respective cut-points in the entire cohort and the carboplatin/paclitaxel cohort. For example, patients with TM-V50 ≥ 1.5% had a 2.66-fold increase (95% CI 1.26–5.80, *p* = 0.012) in the odds of HT3+ for the entire cohort and a 2.68-fold increase (95% CI 1.07–6.97, *p* = 0.038) in the carboplatin/paclitaxel cohort after adjusting for confounders.

### Predictors of Hematologic Toxicities: Total Marrow Sub-Sites

Next, we performed univariate analyses of each TM sub-site in both the entire cohort and carboplatin/paclitaxel cohorts with results summarized in Tables 4, 6. While several sternum dosimetric parameters were associated with HT3+ in the entire cohort on univariate analysis (Table 4), none were associated or borderline-associated with HT3+ in the carboplatin/paclitaxel cohort (Table 6). Therefore, we focus the rest of this section on the VB and ribs dosimetric parameters.

On multivariate analysis, the VB-mean dose (OR = 1.06, *p* = 0.032), VB-V30 (OR = 1.15, *p* = 0.010), VB-V40 (OR = 1.19, *p* = 0.002) and VB-V50 (OR = 1.32, *p* = 0.013) were associated with higher rates of HT3+ in the entire cohort (Table 8) while only VB-V40 (OR = 1.16, *p* = 0.044) remained significant in the carboplatin/paclitaxel cohort (Table 9). The optimal cut-point for VB-V40 was 29% resulting in odds-ratios of 3.58 (95% CI 1.60–8.04, *p* = 0.002) and 3.55 (95% CI 1.36–9.26, *p* = 0.010) for patients with VB-V40 ≥ 29% vs. VB-V40 < 29% in the entire cohort and carboplatin/paclitaxel cohorts, respectively.

**Table 8:** Multivariate logistic regression analysis of factors associated with grade ≥ 3 hematologic toxicities using the VB and rib sub-sites only in the entire cohort.

**Model**	**OR (95% CI)**	***p*-value**
**Model 1: VB-mean**		
VB-mean (per 1 Gy)	1.06 (1.01–1.12)	0.032
Age	1.04 (1.00–1.08)	0.032
Male vs. Female	0.50 (0.18–1.37)	0.178
C/T vs. Other	3.20 (1.46–7.39)	0.005
**Model 2: VB-V30**		
VB-V30 (continuous)	1.15 (1.04–1.28)	0.010
Age	1.04 (1.01–1.08)	0.024
Male vs. Female	0.47 (0.17–1.32)	0.153
C/T vs. Other	3.54 (1.59–8.40)	0.003
**Model 3: VB-V40**		
VB-V40 (continuous)	1.19 (1.06–1.34)	0.004
Age	1.04 (1.01–1.08)	0.028
Male vs. Female	0.58 (0.20–1.60)	0.289
C/T vs. Other	3.76 (1.67–9.10)	0.002
VB-V40 ≥ 29%	3.58 (1.60–8.04)	0.002
Age	1.04 (1.01–1.08)	0.018
Male vs. Female	0.47 (0.17–1.34)	0.160
C/T vs. Other	3.74 (1.62–8.64)	0.002
**Model 4: VB-V50**		
VB-V50 (continuous)	1.32 (1.03–1.73)	0.013
Age	1.04 (1.00–1.08)	0.040
Male vs. Female	0.54 (0.20–1.45)	0.221
C/T vs. Other	3.09 (1.42–7.08)	0.006
**Model 5: Rib-mean**		
Rib-Mean (per 1 Gy)	1.25 (1.07–1.46)	0.005
Age	1.04 (1.00–1.08)	0.037
Male vs. Female	0.47 (0.17–1.28)	0.140
C/T vs. Other	3.25 (1.47–7.62)	0.005
Rib-Mean ≥ 9.4 Gy	5.67 (2.42–14.54)	<0.001
Age	1.05 (1.01–1.09)	0.014
Male vs. Female	0.36 (0.12–1.02)	0.058
C/T vs. Other	4.37 (1.88–10.92)	0.001
**Model 6: Rib-V20**		
Rib-V20 (continuous)	1.41 (1.08–1.88)	0.014
Age	1.04 (1.00–1.08)	0.036
Male vs. Female	0.40 (0.14–1.11)	0.082
C/T vs. Other	3.25 (1.48–7.53)	0.004
Rib-V20 ≥ 16%	3.65 (1.69–8.23)	0.001
Age	1.04 (1.00–1.08)	0.058
Male vs. Female	0.40 (0.14–1.11)	0.080
C/T vs. Other	3.17 (1.43–7.39)	0.006
**Model 7: Rib-V30**		
Rib-V30 (continuous)	2.20 (1.44–3.52)	0.001
Age	1.04 (1.01–1.08)	0.024
Male vs. Female	0.46 (0.16–1.27)	0.135
C/T vs. Other	5.05 (2.10–13.34)	0.001
Rib-V30 ≥ 5%	6.82 (2.89–17.69)	<0.001
Age	1.04 (1.00–1.09)	0.021
Male vs. Female	0.46 (0.16–1.31)	0.146
C/T vs. Other	5.38 (2.21–14.41)	<0.001

*OR, odds ratio; CI, confidence interval; VB, vertebral body; C/T, carboplatin/paclitaxel; Vx, volume of structure receiving ≥x Gy*.

**Table 9:** Multivariate logistic regression analysis of factors associated with grade ≥ 3 hematologic toxicities using the VB and rib sub-sites only in the carboplatin/paclitaxel cohort.

**Model**	**OR (95% CI)**	***p*-value**
**Model 1: VB-V40**		
VB-V40 (continuous)	1.16 (1.01–1.34)	0.044
Age	1.06 (1.01–1.11)	0.013
VB-V40 ≥ 29%	3.55 (1.36–9.26)	0.010
Age	1.06 (1.01–1.11)	0.010
**Model 2: Rib-mean**		
Rib-mean (per 1 Gy)	1.26 (1.02–1.58)	0.040
Age	1.06 (1.02–1.11)	0.013
Rib-Mean ≥ 9.4 Gy	2.68 (1.07–6.97)	0.038
Age	1.06 (1.01–1.11)	0.014
**Model 3: Rib-V20**		
Rib-V20 (continuous)	1.43 (1.02–2.08)	0.047
Age	1.05 (1.01–1.10)	0.013
Rib-V20 ≥ 16%	3.34 (1.30–8.97)	0.014
Age	1.05 (1.01–1.10)	0.025
**Model 4: Rib-V30**		
Rib-V30 (continuous)	1.99 (1.13–3.76)	0.024
Age	1.05 (1.01–1.10)	0.021
Rib-V30 ≥ 5%	3.72 (1.42–10.37)	0.009
Age	1.05 (1.01–1.10)	0.017

*OR, odds ratio; CI, confidence interval; VB, vertebral body; Vx, volume of structure receiving ≥x Gy*.

Similarly, multivariate analysis revealed that rib-mean (OR = 1.25, *p* = 0.005), rib-V20 (OR = 1.41, *p* = 0.014), and rib-V30 (OR = 2.20, *p* = 0.001) were significantly associated with HT3+ in the entire cohort (Table 8). Those 3 variables (rib-mean, rib-V20, and rib-V30) remained significantly associated with HT3+ in the carboplatin/taxol cohort (Table 9).

The rib-mean dose (OR = 1.20, *p* = 0.013), rib-V10 (OR = 1.16, *p* = 0.046), and rib-V30 (OR = 1.59, *p* = 0.013) were associated with higher rates of HT3+ in the entire cohort while rib-V20 (OR = 1.26, *p* = 0.071) was borderline associated with HT3+. In the carboplatin/paclitaxel cohort, rib-mean (OR = 1.24, *p* = 0.046), rib-V20 (OR = 1.43, *p* = 0.038), and rib-V30 (OR = 2.03, *p* = 0.015) were significantly associated with HT3+. The optimal cut-points for rib-mean, rib-V20, and rib-V30 were 9.4 Gy, 16 and 5%, respectively.

We performed an exploratory direct comparison of the significant VB and rib parameters. Table 10 demonstrates the multivariate analyses incorporating the optimal cutpoints for VB-V40 and rib-mean, rib-V20, or rib-V30 for the entire cohort and the carboplatin/paclitaxel cohort. For the entire cohort, the rib dosimetric parameter slightly out-performed (smaller *p*-values) the VB-V40 while the VB-V40 generally outperformed the rib parameters in the carboplatin/taxol cohort.

**Table 10:** Multivariate analyses including the VB and rib dosimetric parameters in the entire cohort and carboplatin/paclitaxel cohorts.

	**All patients**	**C/T cohort**
	**OR (95% CI), *p*-value**	**OR (95% CI), *p*-value**
VB-V40 ≥ 29%	2.42 (1.00–6.06), *p =* 0.053	2.49 (0.84–7.64), *p =* 0.103
Rib-Mean ≥ 9.4 Gy	2.37 (1.01–5.67), *p =* 0.049	2.07 (0.68–6.33), *p =* 0.197
Age	1.04 (1.00–1.08), *p =* 0.035	1.06 (1.02–1.11), *p =* 0.013
Male vs. Female	0.56 (0.19–1.62), *p =* 0.280	N/A
C/T vs. Other	3.71 (1.62–9.05), *p =* 0.003	N/A
VB-V40 ≥ 29%	2.91 (1.29–6.88), *p =* 0.012	2.71 (1.00–7.64), *p =* 0.053
Rib-V20 ≥ 16%	3.01 (1.35–6.93), *p =* 0.008	2.46 (0.89–6.94), *p =* 0.083
Age	1.04 (1.01–1.08), *p =* 0.031	1.06 (1.01–1.11), *p =* 0.175
Male vs. Female	0.39 (0.13–1.13), *p =* 0.082	N/A
C/T vs. Other	3.93 (1.71–9.63), *p =* 0.002	N/A
VB-V40 ≥ 29%	2.09 (0.86–5.18), *p =* 0.105	2.50 (0.88–7.25), *p =* 0.086
Rib-V30 ≥ 5%	5.23 (2.10–14.16), *p =* 0.001	2.59 (0.89–7.76), *p =* 0.082
Age	1.05 (1.01–1.09), *p =* 0.015	1.06 (1.02–1.11), *p =* 0.012
Male vs. Female	0.44 (0.14–1.29), *p =* 0.135	N/A
C/T vs. Other	5.86 (2.37–16.13), *p* < 0.001	N/A

*OR, odds ratio; CI, confidence interval; VB, vertebral body; Vx, volume of structure receiving ≥x Gy; C/T, carboplatin/paclitaxel; N/A, not applicable*.

## Discussion

In this study, we found that increasing TM radiation dose is associated with the development of acute HT3+ in esophageal cancer patients treated with CRT. In our NTCP analysis for the entire cohort, we found that the *n* value in the LKB model was close but not exactly equal to 1 *(n* = 0.70) and in the carboplatin/paclitaxel cohort, our results demonstrated that *n* = 0.30. These findings imply that the TM structure is not exactly a parallel organ in the way we defined it for this study. Nonetheless, TM-mean dose as well as many of the other dosimetric parameters had a strong association with the development of HT3+.

The observation that the LKB model resulted in a value of *n* = 0.70 for the entire cohort and *n* = 0.30 for the carboplatin/paclitaxel cohort was unexpected. A previous study of patients receiving thoracic CRT for lung cancer used the thoracic VBs as a surrogate for bone marrow found that the *n* = 1 when the LKB model was applied to the data set (7). In that study, VB was defined as the T1-T10 vertebral bodies, which was slightly different than the VB definition in this study, where we included T1-L1 (given that distal esophageal and GE junction tumors would result in more radiation exposure to the lower thoracic vertebrae, as well as L1). We did see that incorporating the ribs and sternum into the structure TM increased the *n* to closer to 1. In addition, previous studies of pelvic bone marrow (lumbosacral spine beginning from the top of L5, the ilium, and the low pelvic bones) in patients receiving CRT for anal/gynecologic malignancies have also found a value of *n* = 1 on LKB modeling (10, 12, 13). A value of *n* = 1 makes physiologic sense as the interpretation is that the marrow is composed of functional subunits, similar to organs such as the lung and liver, and that it is the mean radiation dose to the marrow that is the driver of toxicity. In our study, particularly in the more uniform group of patients treated with carboplatin/paclitaxel, the mean TM dose was not strongly associated with development of HT3+, which at least is consistent with the LKB model, in which *n* = 0.30. The reason for the discrepant *n* value we found could be due to a multitude of factors including an insufficient sample size to detect the mean dose as a significant predictor of HT3+, an incorrect definition of the TM structure, and the variability in chemotherapy regimens. We tried to account for some of these factors with subgroup analyses (e.g., the carboplatin/paclitaxel group only), but ultimately, validation of these results in a separate and ideally prospectively followed cohort would be needed.

Another interesting component of the NTCP modeling occurred when the *n* value was defined as 1, and optimization of the LKB model was done for only the TD_50_ and *m* values. Here we see the impact of chemotherapy on the LKB model. In patients treated with carboplatin/paclitaxel, the TD_50_ = 14.0 Gy and the slope parameter *m* = 1.16, which results in a shallow, slowly rising NTCP curve. For patients treated with other chemotherapy regimens, TD_50_ = 19.6 Gy and *m* = 0.42 (steep, faster rise). Consistent with the results of the LKB models, significant differences in acute HT3+ were seen in patients depending on the concurrent chemotherapy regimen. On multivariate analysis, treatment with carboplatin/paclitaxel, the most common regimen at our institution, was the variable most strongly associated with development of HT3+. These findings lead to the hypothesis that it may be more important to keep radiation dose to the TM at a minimum for patients treated with carboplatin/paclitaxel as opposed to other regimens.

To date, only one other study has examined the impact of radiation dose to bony structures in the thorax in patients receiving CRT for EC (9). This study included 41 patients all treated with cisplatin/5FU and IMRT to a dose of 41.2–43.2 Gy to a large field with a simultaneous integrated boost to the gross disease to 46–48 Gy. The VB was defined as T1-T12 and radiation to the ribs, sternum, scapula and clavicle were also assessed. The endpoints analyzed included grade ≥ 3 WBC or grade ≥ 2 ANC. The authors found that higher radiation dose to the VB (mean dose and V5-V30) and rib (mean dose and V5-V30) were associated with higher rates of grade 3 WBC toxicity, and that the VB dose had the strongest association with hematologic toxicity. The study suggested constraining the VB mean dose <35.9 Gy, V20 < 70%, and V10 < 77% for patients receiving concurrent cisplatin/5FU. Irradiation of the VBs has also been studied in the setting of lung cancer. Barney et al. suggested constraining the VB to mean dose ≤23Gy, V5 ≤ 65%, V10 ≤ 60%, and V20 ≤ 50% may decrease acute HT in patients receiving CRT for NSCLC (7). Deek et al. also noted the relationship between VB radiation and leukopenia, recommending a mean VB doses of ≤ 23.9 Gy, V20 ≤ 56.0%, and V30 ≤ 52.1% (8). While the radiation therapy techniques and doses, chemotherapy regimens, and endpoints used in these studies vary from each other and the current study, the important message is that radiation therapy to the bony marrow in the thorax (particularly the VB and ribs) contributes to HT.

Contouring the TM in the thorax is tedious. There are significant heterogeneities in Hounsfield Units in the cortex and the marrow of bony structures, which poses challenges for auto-contouring features in treatment planning software. This is particularly true for each individual rib as it courses from the costovertebral junction to the costo-sternal junction. Thus, we were interested in analyzing whether any of the other sub-sites (especially the sternum or VB) alone could serve as a surrogate for the TM as a dose-limiting structure in an effort to reduce hematologic toxicities. While none of the sternum dosimetric parameters were associated with HT3+ in the carboplatin/paclitaxel cohort, there were VB dosimetric parameters associated with HT3+ in this group as well as the entire cohort, particularly VB-V40. Of course, the rib mean dose, rib-V20 and rib-V40 were also associated with HT3+. In direct comparison of VB-V40 and rib dosimetric parameters, the rib parameters appeared to be slightly more important on multivariate analysis in the entire cohort, but not in the more uniform cohort of patients that received carboplatin/paclitaxel. Given the significant amount of time it currently takes to contour the ribs, prospectively investigating whether limiting the VB-V40 (or other VB parameters) is appealing. If validated, the more pragmatic approach of contouring VBs would have a greater impact on saving time in busy, routine clinical practices.

This study is subject to numerous limitations. First and foremost, this is a retrospective analysis from a single institution and all of the results can be viewed as only hypothesis-generating. However, these data could serve as a solid starting point for a prospective study, as a secondary analysis of currently ongoing or recently completed cooperative group clinical trials involving chemoradiation in esophageal cancer patients, or as baseline data to be externally validated in an independent data set. In addition, our definition of one of the TM sub-sites, the VB structure (T1-L1) is different from that of Lee et al. (T1-T12) which is also different from the lung cancer studies (T1-T10). We considered these differences at the study outset but felt strongly that the VB structure should include L1 due to the frequent radiation exposure of L1 in patients receiving CRT for lower thoracic or gastroesophageal junction tumors, which comprised 84% of our patient population. Ideally, the structure-at-risk should be defined as the entire bone marrow for each patient, but this approach is neither practical nor possible in routine practice. Lastly, the bony structures contoured on CT imaging are used as a surrogate for active bone marrow, but associations between radiation dose to the TM and HT3+ may be stronger if functional imaging techniques were used to define active bone marrow as has been done in pelvic malignancies (14–17).

Nonetheless, these data provide a starting point for future prospective evaluation. Currently, we have a single arm phase II trial under development for esophageal cancer patients undergoing chemoradiation with IMRT and weekly carboplatin/paclitaxel. We will assess the feasibility of constraining the VB-V40 < 29% while not affecting our lung or heart dosimetric parameters. The study will be powered to reduce the estimated HT3+ toxicity rate from 50% to <35%. If this trial meets its primary endpoint, then a randomized trial of IMRT vs. 3DCRT in this patient population is warranted.

In conclusion, we found that increasing TM radiation dose was associated with HT3+ in patients with esophageal cancer treated with CRT. Our results showed that radiation dose to sub-sites of the TM, particularly, the ribs and the VB, is also associated with HT3+. In particular, VB-V40 > 29% was associated with a >3.5-fold increased rate of HT3+ in all patients and in the group treated with carboplatin/paclitaxel. From a practical standpoint, limiting dose to the VB (as opposed to the TM or the ribs) may be sufficient to limit HT3+, but further prospective evaluation of these results, as described above, is needed.

## Data Availability

The datasets generated for this study are available on request to the corresponding author.

## Ethics Statement

This study was carried out in accordance with the recommendations of The Ohio State University Institutional Review Board. This was a retrospective study and is therefore exempt from obtaining written informed consent from subjects. The retrospective protocol was approved by the Institutional Review Board of The Ohio State University.

## Author Contributions

DF and JB wrote the manuscript. DF, CB, and JA extracted the data and created the database. AA, JB, and DF performed the statistical analyses. All authors reviewed the data analysis, study conclusions, contributed to the manuscript revision, read, and approved the submitted version.

### Conflict of Interest Statement

The authors declare that the research was conducted in the absence of any commercial or financial relationships that could be construed as a potential conflict of interest.
